# Activated fibronectin-secretory phenotype of mesenchymal stromal cells in pre-fibrotic myeloproliferative neoplasms

**DOI:** 10.1186/s13045-014-0092-2

**Published:** 2014-12-14

**Authors:** Rebekka K Schneider, Susanne Ziegler, Isabelle Leisten, Monica SV Ferreira, Anne Schumacher, Björn Rath, Dirk Fahrenkamp, Gerhard Müller-Newen, Martina Crysandt, Stefan Wilop, Edgar Jost, Steffen Koschmieder, Ruth Knüchel, Tim H Brümmendorf, Patrick Ziegler

**Affiliations:** Division of Hematology, Department of Medicine, Brigham and Women’s Hospital, Harvard Medical School, 1 Blackfan Circle, Boston, MA 02115 USA; Institute of Pathology, University Hospital Aachen, RWTH Aachen University, Pauwelsstrasse 30, Aachen, 52074 Germany; Department of Oncology, Hematology and Stem cell transplantation, University Hospital Aachen, RWTH Aachen University, Pauwelsstrasse 30, Aachen, 52074 Germany; Department of Orthopaedic Surgery, University Hospital Aachen, RWTH Aachen University, Pauwelsstrasse 30, Aachen, 52074 Germany; Department of Biochemistry and Molecular Biology, University Hospital Aachen, RWTH Aachen University, Pauwelsstrasse 30, Aachen, 52074 Germany

**Keywords:** Myeloproliferative neoplasms, Myelofibrosis, Mesenchymal stromal cells, Tissue microarray biomarker, Fibronectin

## Abstract

We characterized bone marrow stromal cells (BMSC) from patients with pre-fibrotic myeloproliferative neoplasms (MPN). MPN-BMSC showed decreased capacity to stimulate the proliferation of colony-forming units of normal hematopoietic stem and progenitor cells and displayed increased matrix remodelling (in particular fibronectin deposition) compared to control BMSC. This finding was confirmed in pre-fibrotic MPN bone marrow biopsies in a tissue microarray (n = 34), which stained positive for fibronectin in the absence of reticulin as a standard myelofibrosis marker. Fibronectin expression correlated significantly with reduced haemoglobin levels in MPN-patients (p = 0.007; R2 = 0.42). Our data show significant cell-intrinsic alterations in MPN-MSC and suggest that Fibronectin expression might be applicable as a biomarker for the identification of early myelofibrotic transformation in reticulin-negative MPN.

## Findings

**Figure 1:**
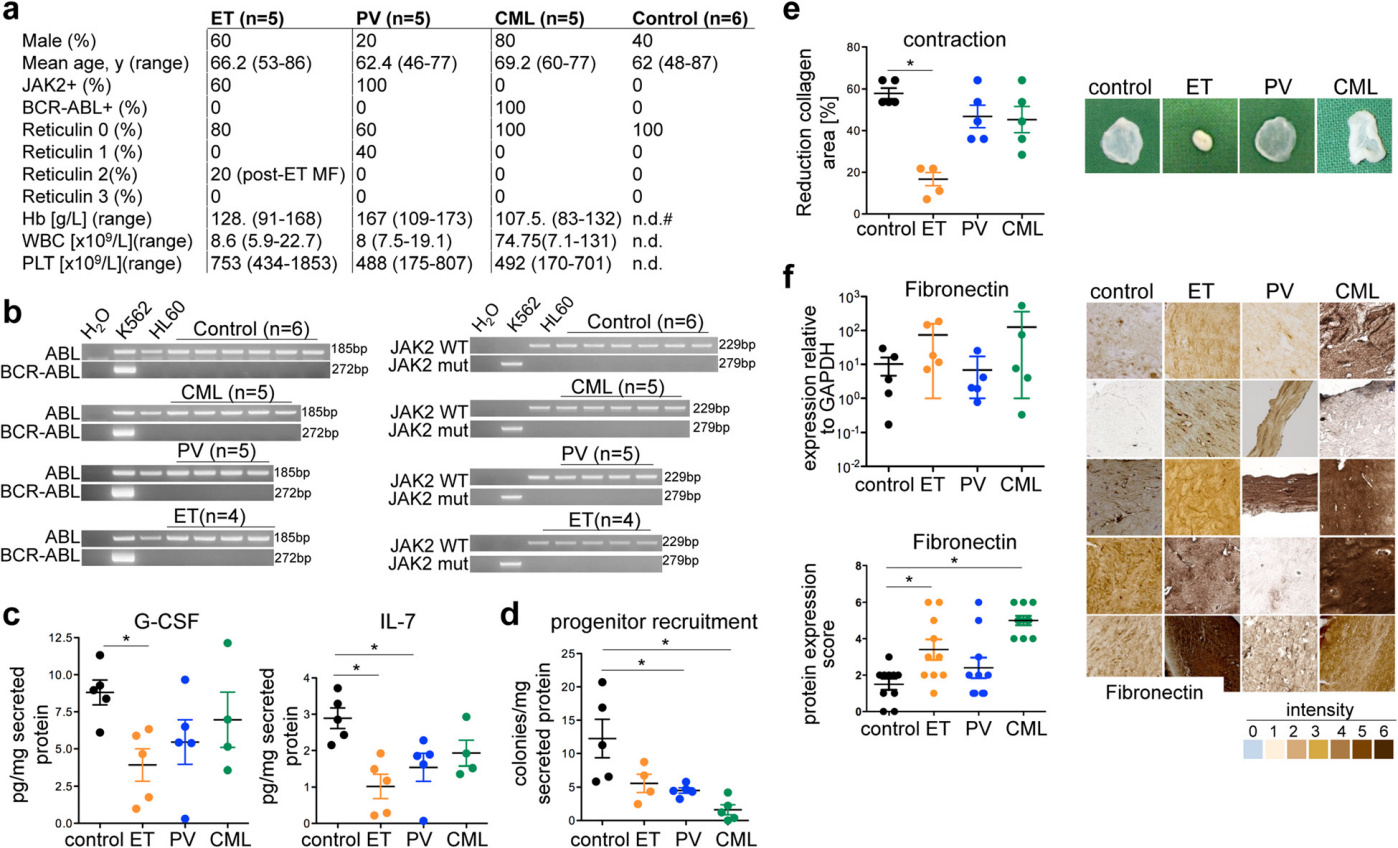
***In vitro*** **screening approach of hematopoiesis-supporting and matrix remodelling capacities of MPN-MSC. (a)** Clinical and laboratory characteristics of MPN-MSC donors and control cases. Reticulin fibrosis was evaluated according to the European consensus. y, years; Hb, haemoglobin levels; WBC, white blood cell count; PLT, platelet count; n.d. not determined. **(b)** BMSCs were isolated from BM of patients with MPN or control donors by plastic adherence. RT-PCR analysis detecting the BCR-ABL gene (left) or JAK2-V617F mutation (right) in cultured BMSC (passage 2). **(c)** Protein levels of G-CSF and IL-7 in BMSC supernatants (passage 2), (mean ± SD, n = 5 each, *p < 0.05). **(d)** CFU activity of 500 sorted human CB CD34^+^ cells in the presence of supernatants from BMSC (mean ± SD, n = 5 for control, PCV and CML; n = 4 for ET, *p < 0.05). **(e)** Contraction of the original collagen gel area (upper panel) in collagen gels after 28 days (mean ± SD, n = 5 each, **p < 0.05). **(f)** Messenger RNA levels of fibronectin in BMSC in 3D collagen after 28 days of culture. Expression levels are normalized against GAPDH. (mean ± SD, n = 5 each, non-signficant). Quantification of fibronectin expression in collagen gels represented in panel 2–6 by 2 independent researchers (according to grading scale 0–6 below panels). (mean ± SD, n = 5 each, *p < 0.05).

It remains elusive if human bone-marrow mesenchymal stromal cells (BMSC) display an intrinsic role in the development of myelofibrosis independent of the malignant clone. We applied a three-dimensional collagen culture-system as an *in vitro*-screening for the functional characterization of BMSC isolated from MPN-patients [1,2]. The assay was designed to be applicable for potential diagnostic and prognostic utility in the investigation of progressive myelofibrosis. The main question of the study was if MPN-MSCs functionally differ from MSCs isolated from healthy donors in (i) their matrix remodelling and (ii) hematopoiesis-supporting capacity. MSC were isolated from patients with ET (n = 4), PV (n = 5), CML (n = 5), all without or only mild myelofibrosis, one patient with post essential thrombocythemia myelofibrosis (ET-MF, n = 1) as well as from control patients (non-MPN, n = 6), (Figure 1a). MSC were isolated from bone marrows by plastic adherence. No significant differences were detected in (1) the number of proliferating mesenchymal precursors (CFU-F), (2) MSC morphology, (3) osteoblastic and adipogenic differentiation or (4) the surface marker pattern of MPN- and control-MSC. In line with previous reports, (RT)-PCR examination of cultured BMSC neither detected the JAK2-V617F mutation nor the BCR-ABL fusion transcript, despite the fact that 3/5 of ET cases and 5/5 of PV were positive for the JAK2-V617F mutation and all CML cases (5/5) were positive for BCR-ABL translocation in peripheral blood cells (Figure 1b).

To characterize the hematopoiesis-supporting capacity of MSC, secreted cytokines and their biological activity were tested. ET-MSC secreted significantly lower levels of G-CSF and IL-7 compared to controls, indicating a defect in the hematopoiesis-supporting capacity (Figure 1c). We therefore evaluated the biological activity of secreted cytokines in myeloid colony-forming unit (CFU) assays using human cord blood (CB) CD34^+^-hematopoietic stem and progenitor cells (HSPCs) [3]. Myeloid CFU-activity significantly decreased when supernatants from PV and CML were used (Figure 1d). These findings indicate that MPN-BMSCs lose their capacity to produce and release functional myeloid differentiation-supporting cytokines.

**Figure 2:**
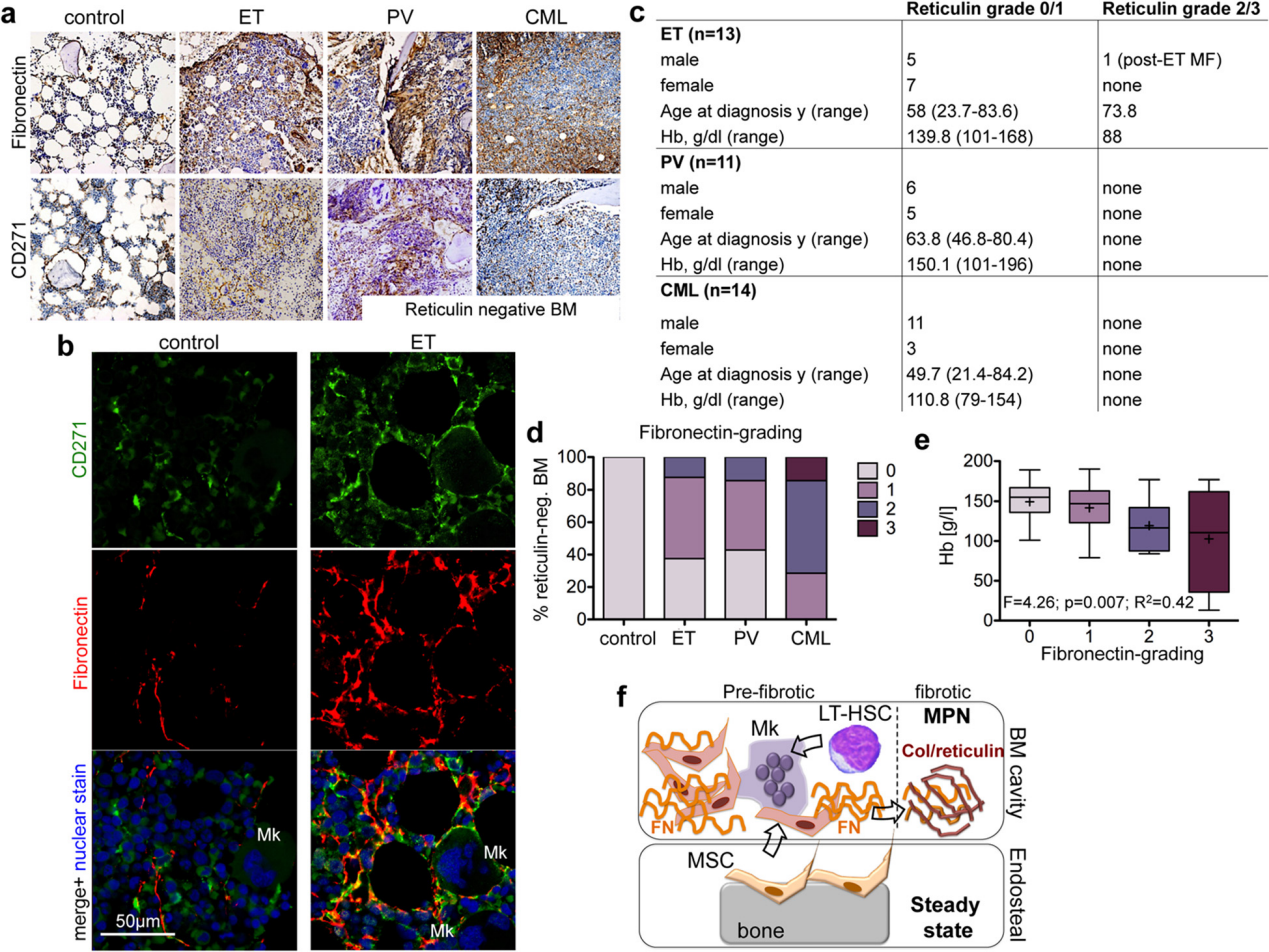
***In vivo*** **validation of the fibronectin-secretory phenotype of MPN-MSC. (a)** Representative Fibronectin and CD271 expression in correlating bone marrow biopsies of MPN-MSC donors. **(b)** Co-localization of fibronectin and CD271 expression by confocal microscopy. CD271 (green, Alexa Fluor 488) and fibronectin (red, Alexa Fluor 555) were analysed separately (panel 1 and 2) and merged (panel 3) with the nuclear counterstain TO-PRO®-3 Iodide (blue, excitation at 633 nm). Cytoplasmatic extensions of CD271^+^ MSC co-localize with Fibronectin, in particular in association to megakaryocytes (Mk). **(c)** Clinical and laboratory characteristics of patient samples included in the tissue microarray (TMA). y, years; Hb, haemoglobin levels. **(d)** Fibronectin grading was adapted to Reticulin grading (Thiele et al.) in reticulin-negative MPN TMA samples. **(e)** Correlation of fibronectin-grading in reticulin-negative graded TMA samples to haemoglobin levels. Box plots show minimal, quartile, median and maximal values in each group, (+) = mean value. **(f)** Schematic depicting the hypothesis that MSC reside in the endosteal (and vascular) niche under steady state conditions and are activated from these niche by dysplastic megakaryocytes (Mk) and the neoplastic clone (LT-HSC). Once activated, MPN-MSC acquire a secretory phenotype that facilitates MSC migration, matrix contraction and provides a provisional matrix for collagen (Col) fibres in more progressed fibrosis.

Further, we tested MPN-BMSCs for their capacity to remodel extracellular matrix (ECM) in the absence of the hematopoietic clone [1,2]. MSC isolated from ET induced a significant reduction of the collagen area accompanied by enhanced matrix stiffening (Figure 1e). We analysed if the contraction potential involves matrix production and scored protein and gene expression of matrix components. A significant up-regulation of the ECM protein fibronectin was detected in ET-MSC by immunohistochemistry (Figure 1f). We next asked if ECM remodelling and the significant differential fibronectin expression can be attributed to MSC in correlating bone marrow biopsies of MSC donors. We applied CD271 for the identification of MSC in bone marrow biopsies as this marker most faithfully identifies the heterogenous stromal cell (MSC) population in the human bone marrow up-to-date [4]. Our first finding was that the distribution of CD271^+^ MSC significantly differed in MPN biopsies compared to the control bone marrow (Figure 2a). In controls, CD271 was mainly expressed in the endosteal and vascular niche [5]. In MPN entities however, CD271^+^ cells were mobilized from these niches and identified in association to dysplastic megakaryocytes (Figure 2a). Our second finding was that fibronectin is expressed in association to CD271 in the hematopoietic niches of the bone marrow in control donors. In striking contrast, fibronectin was diffusely up-regulated in association to expanded stromal cells and identified unbound to the ECM in MPN (Figure 2b) as well as in association to megakaryocytes (Figure 2f).

To validate these findings, we systematically analyzed CD271 and fibronectin in tissue microarrays including ET (n = 13), PV (n = 11), CML (n = 14), PMF (n = 11) and non-MPN controls (n = 17), (Figure 2c). CD271 and fibronectin were evaluated applying a grading scheme similar to the established guidelines for grading myelofibrosis – 0 = no fibrosis and 3 = severe myelofibrosis [6]. Although the majority of MPN cases were classified as myelofibrosis grade 0–1 in reticulin-staining, myelofibrotic transformation was graded as ≥ 1 by Fibronectin-staining in 69% of ET (vs. 7.7% Reticulin-representing one post ET-MF patient), 63% of PV (vs. 0% Reticulin) and 100% CML (vs. 0% Reticulin) cases and by CD271 staining in 54% of ET, 51% PV and 64% CML cases (Figure 2d). In line with the observation of a pre-fibrotic marrow, fibronectin and CD271 expression grade correlated significantly with decreased haemoglobin levels in reticulin-negative biopsies (Figure 2e).

In conclusion, our data reveal an intrinsic defect of MSC in pre-fibrotic MPN resulting in decreased hematopoiesis-supporting capacities and increased ECM remodelling. Our data suggest that fibronectin up-regulation/distribution detects early myelofibrotic changes in the BM of MPN patients and implies clinical and prognostic application in different myeloproliferative (Ph^−^ and Ph^+^) neoplasms.

## References

[1] Kramann R, Couson SK, Neuss S, Kunter U, Bovi M, Bornemann J, Knuchel R, Jahnen-Dechent W, Floege J, Schneider RK (2011). Exposure to uremic serum induces a procalcific phenotype in human mesenchymal stem cells. Arterioscler Thromb Vasc Biol.

[2] Schneider RK, Puellen A, Kramann R, Raupach K, Bornemann J, Knuechel R, Perez-Bouza A, Neuss S (2010). The osteogenic differentiation of adult bone marrow and perinatal umbilical mesenchymal stem cells and matrix remodelling in three-dimensional collagen scaffolds. Biomaterials.

[3] Manz MG, Miyamoto T, Akashi K, Weissman IL (2002). Prospective isolation of human clonogenic common myeloid progenitors. Proc Natl Acad Sci U S A.

[4] Tormin A, Li O, Brune JC, Walsh S, Schutz B, Ehinger M, Ditzel N, Kassem M, Scheding S (2011). CD146 expression on primary nonhematopoietic bone marrow stem cells is correlated with in situ localization. Blood.

[5] Lo Celso C, Fleming HE, Wu JW, Zhao CX, Miake-Lye S, Fujisaki J, Cote D, Rowe DW, Lin CP, Scadden DT (2009). Live-animal tracking of individual haematopoietic stem/progenitor cells in their niche. Nature.

[6] Thiele J, Kvasnicka HM, Facchetti F, Franco V, van der Walt J, Orazi A (2005). European consensus on grading bone marrow fibrosis and assessment of cellularity. Haematologica.

